# Plant Secondary Metabolites: The Weapons for Biotic Stress Management

**DOI:** 10.3390/metabo13060716

**Published:** 2023-05-31

**Authors:** Jameel M. Al-Khayri, Ramakrishnan Rashmi, Varsha Toppo, Pranjali Bajrang Chole, Akshatha Banadka, Wudali Narasimha Sudheer, Praveen Nagella, Wael Fathi Shehata, Muneera Qassim Al-Mssallem, Fatima Mohammed Alessa, Mustafa Ibrahim Almaghasla, Adel Abdel-Sabour Rezk

**Affiliations:** 1 Department of Agricultural Biotechnology, College of Agriculture and Food Sciences, King Faisal University, Al-Ahsa 31982, Saudi Arabia; wshehata@kfu.edu.sa (W.F.S.); arazk@kfu.edu.sa (A.A.-S.R.); 2 Department of Life Sciences, CHRIST (Deemed to be University), Bangalore 560 029, Karnataka, India; rashmi.r@res.christuniversity.in (R.R.); varsha.toppo@res.christuniversity.in (V.T.); cholepranjali.bajrang@res.christuniversity.in (P.B.C.); akshatha.b@res.christuniversity.in (A.B.); wudali.sudheer@res.christuniversity.in (W.N.S.); 3 Department of Food Science and Nutrition, College of Agriculture and Food Sciences, King Faisal University, Al-Ahsa 31982, Saudi Arabia; mmssallem@kfu.edu.sa (M.Q.A.-M.); falissa@kfu.edu.sa (F.M.A.); 4 Department of Arid Land Agriculture, College of Agriculture and Food Sciences, King Faisal University, Al-Ahsa 31982, Saudi Arabia; malmghaslah@kfu.edu.sa; 5 Plant Pests, and Diseases Unit, College of Agriculture and Food Sciences, King Faisal University, Al-Ahsa 31982, Saudi Arabia; 6 Department of Virus and Phytoplasma, Plant Pathology Institute, Agricultural Research Center, Giza 12619, Egypt

**Keywords:** biotic stress, plant secondary metabolites, feeding deterrents, aposematic signals, metabolomics engineering, companion farming, biopesticides

## Abstract

The rise in global temperature also favors the multiplication of pests and pathogens, which calls into question global food security. Plants have developed special coping mechanisms since they are sessile and lack an immune system. These mechanisms use a variety of secondary metabolites as weapons to avoid obstacles, adapt to their changing environment, and survive in less-than-ideal circumstances. Plant secondary metabolites include phenolic compounds, alkaloids, glycosides, and terpenoids, which are stored in specialized structures such as latex, trichomes, resin ducts, etc. Secondary metabolites help the plants to be safe from biotic stressors, either by repelling them or attracting their enemies, or exerting toxic effects on them. Modern omics technologies enable the elucidation of the structural and functional properties of these metabolites along with their biosynthesis. A better understanding of the enzymatic regulations and molecular mechanisms aids in the exploitation of secondary metabolites in modern pest management approaches such as biopesticides and integrated pest management. The current review provides an overview of the major plant secondary metabolites that play significant roles in enhancing biotic stress tolerance. It examines their involvement in both indirect and direct defense mechanisms, as well as their storage within plant tissues. Additionally, this review explores the importance of metabolomics approaches in elucidating the significance of secondary metabolites in biotic stress tolerance. The application of metabolic engineering in breeding for biotic stress resistance is discussed, along with the exploitation of secondary metabolites for sustainable pest management.

## 1. Introduction

Plants are exposed to a wide variety of environmental stresses, such as biotic and abiotic stresses, that affect the productivity of agricultural crops. Abiotic stress is caused by variations in physical or chemical factors such as droughts, salinity, floods, heavy metals, extreme temperature, etc. [1]. On the other hand, biotic stress is brought about by living organisms such as arachnids, bacteria, fungi, herbivores, insects, nematodes, oomycetes, viruses, and weeds [2]. Plants die due to deprivation of nutrients, diseases, and infections caused by biotic agents, resulting in major pre- and postharvest losses [3]. The fungal parasites can kill the host cell by releasing toxin (necrotrophic) or feed on living host cells (biotrophic), causing cankers, vascular wilts, and leaf spots in affected plants [4,5,6]. Nematodes, which feed on plants, are the main culprits behind soil-borne illnesses that result in stunted plant growth, nutrient deficiencies, and wilting [7,8]. Likewise, viruses are known to induce chlorosis and stunting caused by systemic and local damage [9]. On the other hand, insects and mites feed on the plants by sucking and piercing and lay their eggs on them and also serve as carriers of pathogenic viruses and bacteria [6].

Due to their sessile nature and lack of an adaptive immune system, unlike animals, plants cannot flee from stress and hence have developed sophisticated strategies in order to survive biotic and abiotic stresses [10]. The sort of biotic stress that can be applied to crop plants depends on the environment in which they are grown and their ability to withstand the biotic stress. The defense mechanism would be either constitutive (pre-existing) or induced. The constitutive defensive mechanisms include internal and external structural barriers such as wax and cuticle, which deter air-bone spores or propagules due to the negative charge developed. Suberization of the epidermis and lenticels makes it more resistant to pathogens. The first line of defense against insect herbivores is provided by trichomes. Organic compounds such as amino acids, sugars, organic acids, enzymes, glycosides, and calcium oxalate crystals also take part in the defense response to biotic stress [11]. The genetic code housed in plants regulates the defense mechanisms that are resistant to various biotic stressors. Biotic stress can also induce structural changes such as gum deposition, suberization, and the formation of abscission layers and tyloses by the protrusion of the xylem [12].

Secondary metabolites are compounds that help a plant survive in a competitive environment and are not a part of the plant’s regular development and progress, unlike primary metabolites [13]. According to the biochemical co-evolutionary arms-race theory, herbivore resistance mechanisms developed as a result of the emergence of plant secondary metabolites in response to herbivore pressure [14]. There are two ways for plants to escape getting destroyed. First, plants can prevent themselves from being picked for oviposition or herbivory by releasing compounds that repel oviposition-inducing herbivores and entice parasitic and predatory insects by eradicating plant-feeding insects, which will lessen additional harm. Second, plants can generate substances that increase herbivore fatality [15]. In place of a complex immune system with specialized antibodies, plants use antibiotic secondary metabolites (SMs) in their innate immune system to protect themselves against microorganisms [16].

In the plant kingdom, more than 2,140,000 secondary metabolites have been reported to date. These substances are a hugely varied category of organic materials produced by a wide range of organisms, fungi, bacteria, algae, and animals. The repertoire of secondary metabolites such as alkaloids, phenolic compounds, and terpenoids are classified based on their biosynthetic origin, function, and structure. There are five primary categories of secondary metabolites: alkaloids, fatty acid-derived compounds and polyketides, enzyme cofactors, non-ribosomal polypeptides, terpenoids, and steroids [17]. Since the secondary metabolites are produced by plants in response to biotic stress, the biotic stressors are used as elicitors to enhance secondary metabolite production [18].

## 2. Methodology of Review

In conducting the literature survey for this article, we relied on scientific resources such as PubMed, ScienceDirect, Scopus, ResearchGate, and Google Scholar, with a focus on ScienceDirect and Scopus. The keywords used for collecting literature were “biotic stress”, “plant secondary metabolites”, and “defense mechanism”. The chronological period in which the papers were published was not considered during the literature survey and review writing process because the primary focus was on noteworthy works that were picked for the topics covered. All relevant information was examined in the selected articles.

## 3. Major Plant Secondary Metabolites Involved in Biotic Stress Tolerance

**Figure 1:**
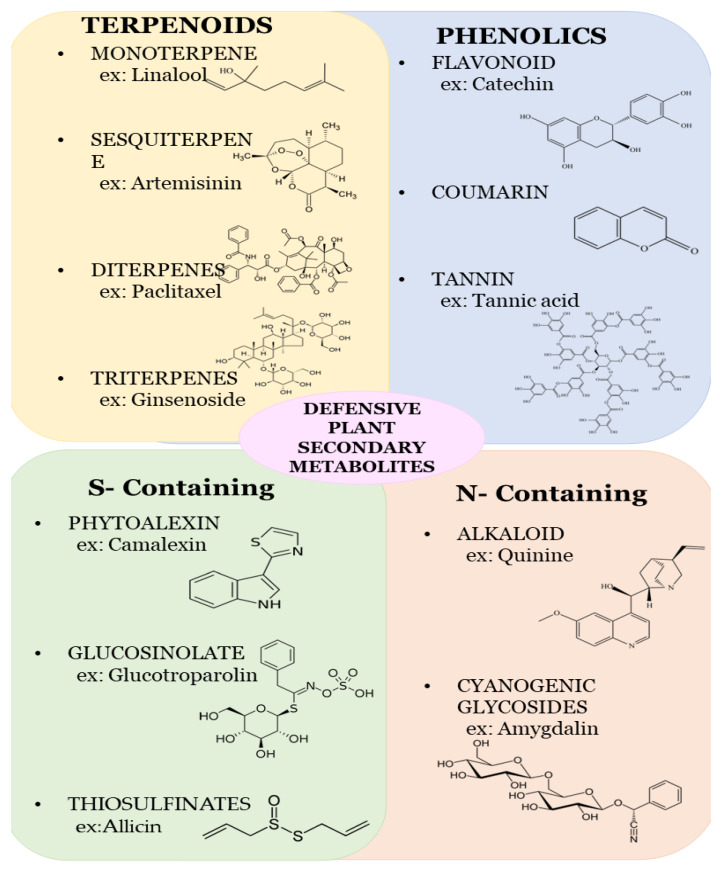
Chemical structures of some important plant secondary metabolites.

Plant secondary metabolites are categorized into terpenoids (such as saponin), phenolics (such as flavones, lignin, isoorientin, tannin, flavonoids, and glyceollin), and nitrogen compounds (such as sinigrin and dhurrin). Different secondary metabolites show different metabolisms, which help to suppress the growth and development of herbivores [19]. Phenolic metabolites with volatile compounds repel herbivores and protect the plant. The detailed study of plant metabolites, their synthesis, and their function is illustrated below (Figure 1).

### 3.1. Terpenes

Terpenes/terpenoids are the largest diversified chemical group of secondary metabolites, which contain more than 22,000 compounds [20]. Terpenes are present in almost all plants. Usually, 5-C isopentanoid units play an essential role in the formation of terpenes. According to the number of isoprene units, terpenes are further classified [21]. Terpenes are commonly synthesized from isomer dimethylallyl diphosphate (DMAPP) and isopentenyl diphosphate (IDP). These blocks are synthesized with two different pathways: the pyruvate-derived plastidial 2-C-methyl-D-erythritol-4-phosphate (MEP) pathway and the acetyl-CoA-derived cytosolic mevalonate (MVA) pathway [22].

In plants, terpenes have both physiological and ecological functions related to plant hormones (gibberellin, abscisic acid), insecticides, allelopathy, and insect pollination. Terpenes such as menthol, camphor, pyrethrins, artemisinins, and farnesol play an important role against protozoa, bacteria, and fungi [20]. A common example, capsaicin, which is present in pepper chilly, shows bactericidal properties. Terpenes generally perform several functions in plants. They act as a defense molecule against pathogens and herbivores, plant growth regulators, and compounds that influence (indirectly or directly) the development and growth of neighboring plants. As per a few reports, hemiterpene increases photosynthesis and thermo-tolerance in some plant species. It has been shown that when oak (*Quercus ilex*) leaves are fumigated with monoterpenes, the thermo-tolerance of the plant increases [23]. Terpene derivatives such as sterol work as an important component in cell membranes by stabilizing the interaction with phospholipids [24]. At the time of photosynthesis, volatile gas such as hydrocarbon isoprene (C5H8) is produced, which avoids damage to the cell membrane from extreme light or temperature conditions and protects it. Tetraterpenes such as carotenoids (orange, red, and yellow pigments) work as an accessory pigment in photosynthesis. Tetraterpenes protect photosynthetic tissues from photooxidation as well [23]. Carotenoids prevent the synthesis of singlet oxygen by extinguishing the chlorophyll molecules from the triplet state [25]. Terpenes such as plastoquinone and ubiquinone act as electron carriers. Carotenoids are precursors of abscisic acid, which regulates the stress and developmental responses in plants. Terpenes are present in almost all plants. Usually, 5-C isopentanoid units play an essential role in the formation of terpenes. According to the number of isoprene units, terpenes are further classified [21]. Table 1 illustrates the classification of terpenes based on the number of isoprene units they contain.

### 3.2. Sulfur-Containing Secondary Metabolites

Sulfur-containing metabolites protect plants from pathogenic microbes either by acting as phytoalexins or as phytoanticipants. Glucosinolates, thiosulfinates (such as allicin, which is generated from cysteine sulfoxides), and antimicrobial peptides (such as defensins and thionins) are examples of sulfur-containing secondary metabolites [20]. Most of them are volatile in nature, acrid in taste, or obnoxious in smell. These secondary metabolites are classified into two different classes according to their path of synthesis. In the first group, hydrolysis of the myrosinase enzyme results in formation of glucosinolate. Members of the crucifereae family show this pathway. Cabbage, broccoli, and nasturtium are examples of the glucosinolate–myrosinase pathway. In the second group of sulfur-containing secondary metabolites, hydrolysis of the alliinase enzyme results in the formation of alliin, which is commonly found in the genus Allium. Garlic (*Allium sativum*), leeks (*Allium porrum*), and onion (*Allium cepa*) are examples of the alliin–alliinase pathway [21]. These two pathways are evolved in herbivore defense and avoid pathogenic attack [28]. Table 2 represents examples of various sulfur-containing secondary metabolites.

#### 3.2.1. Glucosinolates (GSLs)

Glucosinolates are sulfur-containing phytoanticipants in which the β-thioglucose unit is present. They are low molecular mass and hold glycosides in the plant. Moreover, glucosinolates show resistance in higher plants to predators, parasites, and competitors. The breakdown of end products shows a protective nature in the volatile form of the substance. It is effective as a repellent or lethal toxin; for example, there are mustard oil glycosides in Cruciferae and allyl sulfoxides in Allium [29]. Garlic, onion, radish, and mustard show specific flavors caused by these substances. Allium sulfides are mainly mono- or disulfide alkyls. Glucosinolates are distributed widely in the Cruciferae and a few related plant families such as Resedaceae, Moringaceae, Capparaceae, and Tovariaceae [30]. In Papaveraceae, the absence of mustard oil glycosides shows very good chemotaxonomic evidence for the differentiation of the Papaveraceae in other plant orders. Nearly 120 side chains of glucosinolates have been studied. From all these, 16 side chains are commonly discovered in crop plants. Alanine, leucine, tryptophan, isoleucine, valine, phenylalanine, and tyrosine are seven common side chains that correspond to amino acids. Most glucosinolates are aliphatic derivatives (sinigrin and glucocapparin); few have benzyl substituents (sinapine). The glucosinolates biosynthesis pathway is similar to the cyanogenic glucoside pathway, and it is synthesized from amino acids. Glucosinolates are known for antibacterial properties and as attractants to aphids and feeding caterpillars [30].

The plant enzyme myrosinase catalyzes the volatile products from glucosinolates and cleaves sulfur atoms from glucose bonds. Aglycone results in a product that rearranges the loss of chemically reactive substances and sulfate, giving a pungent odor. This includes nitriles, isothiocyanates (R-N=C=S), and thiocyanates. These products are toxic in nature and protect plants from herbivores [31]. With that, these products protect plants from different pathogens such as *Pyrenopeziza brassicae* (leaf spot disease), *Sclerotinia sclerotiorum* (sclerotina stem rot), and *Alternaria brassicae* (alternaria). In healthy plants, substrate glucosinolate (GSL) and the plant enzyme myrosinase are located separately from each other. When plant tissues are damaged, which can be the result of several causes, GSL comes in contact with the myrosinase enzyme and it becomes effective. Cabbage show resistance to *P. parasitica*, whereas oilseed rape and Indian mustard are resistant to *L. maculans* due to the highly combined glucosinolate level. In *Brassica*, GSL breakdown products show the effect on various non-pathogenic organisms. These compounds are known as naturally available fungicides, which effectively manage various pest-harvesting pathogens and diseases related to vegetables and fruits [20]. *Brassica napus* L. is a major oil crop which is found in North America and Europe. In oilseed rape (*Brassica napus* L), it has been shown that the types and amounts of glucosinolates vary with age, developmental status, and plant tissue. In young developing leaves, the amount of glucosinolates is high compared with matured leaves. The accumulation of glucosinolates can be influenced by many factors such as mechanical damage, fungal infection, and insect attack. In some recent studies, methyl jasmonate or salicylic acid treatment strongly persuades glucosinolate accumulation in oilseed rape leaves [32].

In the *Arabidopsis thaliana* plant, biosynthesis of the glucosinolate pathway is majorly studied. In the first step of biosynthesis, the primary amino acid gets converted to aldoxime. This aldoxime gets conjugate with sulfur-donating cysteine, and the formation of a complex takes place. This complex is cleaved by the C-S lyase enzyme, and it results in the formation of the toxic substance thiohydroximate [33]. Glycosylation detoxifies thiohydroximate by using uridine diphosphate glucose; desulfoglucosinolate is produced from thiohydroximate glucosyltransferase [34].

Thiosulfinates are another important phytoanticipant, which are abundant in Allium species. Allicin, a dithiosulfinate, organo sulfur compound, which is present in garlic, giving it the characteristic aroma, shows antifungal, antibacterial, and antiviral activity [35,36].

#### 3.2.2. Sulfur-Containing Phytoalexins

Typically, phytoalexins are crucial elements of a plant’s defenses against bacterial and fungal diseases. The phytoalexins of crucifers are indole alkaloids formed from (S)-tryptophan, and the majority of them contain a sulfur atom derived from cysteine [37]. Examples of sulfur-containing phytoalexins are camalexin, brassinin, and rapalexin A [38,39]. Tryptophan is converted into camalexin by the action of indole-3-acetonitrile (IAN), which subsequently combines with GSH to create GS-IAN. Multiple GSTs (GSTF6, GSTU4) are likely engaged in camalexin biogenesis via facilitating the GS-IAN ligation [40]. The accumulation of camalexins under various fungal and bacterial infections with the involvement of cytochrome P450 enzymes, CYP79B2, and CYP79B3 is well studied in *Arbidopsis* [41,42]. The susceptibility of pad3 (phytoalexin deficient 3) mutant *A. thaliana* to fungal and bacterial pathogens clearly depicts the role of camalexins in biotic stress tolerance [38]. Similarly, *Arabidopsis* plants with overexpressing AtABCG34 genes released higher amounts of camalexin and showed an improved defense response to the pathogen, whereas atabcg34 mutants secreted less camalexin and displayed an improved susceptibility to *A. brassicicola* [43]. Apart from that, elemental sulfur produced by some plants such as tomato, tobacco, cocoa, cotton, and French beans, which develops in response to pathogens, is also considered as an inorganic phytolexin [40].

**Table 2:** Examples of phytoalexins present in different plants with target pathogens.

Metabolite	Category	Plant/Family	Reference
Camalexin	Sulfur-containing phytolexin	Brassicaceae members	[39]
Glycosides	Glucosinolates	Cruciferae	[32]
5-(3-buten-1-ynyl)-2,2′-bithiophene	Dithiophene	Tagetes species	[35]
Allicin	Thiosulfinates	Garlic	[36]

### 3.3. Lectins

Lectins are widely found in different plant species, which are bound to a specific group of sugars. Lectins are ubiquitous, carbohydrate-binding (glyco) proteins that show a protective function against various pests. In legume seeds, it is present abundantly. Lectins show the agglutinating property in a cell due to the presence of multiple binding sites. In a few lectins, a single binding site is present. Lectins such as *wheat germ* agglutinin (WGA) (wheat germ), *Phaseolus hemagglutinin* (PHA) (kidney bean seeds), and *Galanthus nivalis* (snowdrop lectin, GNA) (snowdrop) are studied widely as per their insect toxicity and chemical characteristics. GNA and PHA show specific binding with mannose and alpha-GalNAc, respectively [44]. In transgenic plants such as tobacco, when GNA is expressed in a gene, it shows more efficient protection against aphids. This study proved that lectins could be used for crop tolerance to pests. Chitin and GlcNAc beta(1,4) GlcNAc-specific binding was observed in WGA. WGA protects against toxicity by preventing the production of peritropic membranes in lumen-rich midgut in chitin [44].

Lectins show strong insecticidal potential in herbivore digestive systems by working as an antinutritive. Lectins show stability over a large pH range, and they damage the epithelial lumen membrane, therefore, impeding the absorption and digestion of nutrients. *Galanthus nivalis* L. agglutinin (GNA) is the first plant lectin studied against hemipteran insects for its insecticidal properties. Insects such as lepidopteran, coleopteran, and homopteran work as promising agents [12]. Due to a specific interaction with carbohydrate residue present in the cell membrane, mannose-binding lectins have shown effective results against sucking insects. Table 3 presents a comprehensive list of lectins identified in plants, along with their target insects.

### 3.4. Nitrogen-Containing Secondary Metabolites

Carbon elements contribute nearly 40% to the dry weight of plants, whereas nitrogen elements contribute only 2%. However, these organic substances are present in large numbers in plants. These secondary metabolites include cyanogenic glycosides, alkaloids, and some non-protein amino acids. Ammonia is the first form of nitrogen that is present in plants and produced during nitrogen fixation in plant roots. According to plant physiologists, the universal growth hormone, auxin, is an important nitrogen compound. Meanwhile, alkaloid is the largest known class in plants. These metabolites are mainly known for their anti-herbivore role in plants.

#### 3.4.1. Alkaloids

Generally, alkaloids are basic substances with one or more nitrogen atoms and are usually combined with a cyclic system. Alkaloids are bitter-tasting nitrogenous compounds. Usually, they are crystalline, colorless, and optically active substances. They are the largest class of secondary plant substances and are present in 20% of vascular plants, primarily herbaceous dicots and monocots. Alkaloids are absent in mosses, ferns, and lower plants. Alkaloids such as pyrrolizidine are toxic in nature and help against microbial infection and attack from herbivores. Alkaloids are formed from common amino acids such as tryptophan, tyrosine, aspartic acid, and lysine. Tea, cocoa, and coffee are caffeine-containing alkaloids that are toxic to fungi and insects [20]. In tobacco plants, nicotine alkaloid is found, which is transferred from the root to the leaves of the plant, and finally stored in leaf vacuoles. The Capsicum genus produces capsaicin which demonstrates antimicrobial properties and helps in plant defense mechanisms. Their mode of action affects the nervous system, such as the membrane transporting system, synthesis of protein, and chemical transmitters [49]. Spermine and spermidine (polyamines) play essential roles in the development and growth of plants. In the genus *Lipinus*, alkaloids such as sparteine are found stored in the epidermal cells [24]. Various alkaloids are used as pharmaceuticals, narcotics, and poisons. Plant-based alkaloids such as vinblastine, camptothecin, and vincristine are used as gout suppressants with colchicin, anticancer medicines, and sedatives with scopolamine [21].

Usually, alkaloids are classified into the following groups: (1) non-heterocyclic amines; (2) alkaloids having nitrogen atoms with heterocyclic rings and derived from amino acids; and (3) steroidal alkaloids with nitrogen atoms with heterocyclic rings but not derived from any amino acids. Commonly, alkaloids are classified into 14 groups: pyrrolizidine, pyrrol and pyrrolidine, quinoline, pyridine and piperidine, tropane, aporphine, isoquinoline, indolizidine, terpenoid, norlupinane, indole, purine, steroid, and imidazole [21]. Table 4 presents a compilation of these alkaloids along with specific examples.

#### 3.4.2. Cyanogenic Glycosides

Hydrogen cyanide (HCN) is a lethal chemical that is formed by breaking the cyanogenic glycosides. When herbivores feed on plants, damage to plant tissues takes place, which results in the formation of cyanogenic glycosides. Hydroxynitrile lyases and glycosidases become mixed, and thus cyanogenic glycosides are formed. Cyanogenic glycosides are present in Leguminosae, Gramineae, and Rosaceae families [20]. In cyanogenic glycosides, chemical studies are relatively restricted. Around sixty compounds are characterized fully and have been reported in around 130 families from 2650 plants [44]. Lotaustralin and linamarin are the most common cyanogenic glycosides, which are found together in plants such as *Trifolium repens*, *Linum usitatissimum*, *birdsfoot trefoil*, *flax*, and *Lotus corniculatus* [30]. The seeds of cherry, apricot, almond, and peach show the presence of amygdalin, and *Sorghum bicolor* contains dhurrin. Cyanogenic glycosides show anti-herbivore properties in plant defense mechanisms. In clover, cyanogenic glycosides protect young seedlings from snails and slugs. Cyanide glycosides show a highly toxic nature in living organisms because they demonstrate the ability to prevent the electron transfer system by binding to cytochromes. Therefore, cyanogenic glycosides are known as pest-resistant compounds (Table 5).

### 3.5. Phenolic Compounds

Phenolic chemicals are a large class of secondary metabolites necessary for plant development and survival. They are involved in a wide range of physiological and biochemical processes, including plant defense against biotic and abiotic stresses. These substances range in chemical complexity from simple phenolic acids to complex tannins and lignins. They are defined by the presence of one or more hydroxyl (-OH) groups connected to an aromatic benzene ring [60]. Plants can activate genes involved in the phenylpropanoid pathway in response to herbivore feeding, which results in the manufacture of different phenolic chemicals. By blocking digestive enzymes or attaching to proteins, these substances can have harmful effects on herbivores that impair their ability to grow, develop, and reproduce. The production of particular phenolic chemicals in response to herbivory can also draw the herbivores’ natural predators, giving the plant additional defenses [27]. Phenolic heteropolymer lignin has a crucial role in plant defense mechanisms against pathogens by limiting their entry via increasing the toughness of leaves, reducing the feeding, and reducing the nutritional value of leaves. Polyphenol oxidase and peroxidase are enzymes that catalyze the oxidation of phenolic compounds, resulting in the formation of quinones. Quinones can bind covalently to proteins in herbivorous insects, inhibiting their function and serving as a potential defense mechanism in plants against insect damage. This process is known as phenol oxidation and is an important part of plant defense against herbivores [12].

It has been demonstrated that phenolic chemicals produced by the phenylpropanoid pathway build up in rice plants after pest infestation. Vanillic acid, syringic acid, cinnamic acid, and cinnamic acid derivatives are among the phenolic substances with increased concentrations. These phenolic acids were discovered to be prevalent in pest-infested rice plants [61]. Plant disease resistance is greatly influenced by phenolic chemicals. For instance, to ward off onion smudge disease, *Colletotrichum circinans*-infected onion scales accumulate catechol and protocatechuic acid. Similar to how tomato plants respond to being infected with *Fusarium oxysporum*, the cause of fusarium wilt, they respond by accumulating phenolic chemicals, including ferulic, caffeic, and vanillic acid in recovered leaves and roots. Additionally, the bacterium *Pseudomonas syringae* can alter the phenolic acid composition and improve extracellular phenolic accumulation in *Nicotiana tabacum* [60]. Wheat cultivars that have higher levels of cell-bound and soluble phenolics are less vulnerable to cereal aphids (*Rhopalosiphum padi*) compared with those with lower phenolic concentrations. Strawberry leaves that have a higher constitutive concentration of catechol-based phenolics are more resistant to the two-spotted spider mite (*Tetranychus urticae*), which is attributed to the high concentration of phenolics that suppress mite development in cultivars, especially those with high catechol concentrations. The concentration of phenolics in the bark of American beech trees *(Fagus grandifolia*) remained elevated even six months after being attacked by *Nectaria coccinea var. faginata* [32]. Table 6 showcases the impact of biotic stress on phenolic compounds.

#### 3.5.1. Flavonoids

Flavonoids are a type of phenylpropanoids that act as pigments; they are soluble in water and are stored in the vacuoles of plant cells. There are 12 subgroups of flavonoids, including flavanones, chalcones, stilbenes, aurones, isoflavones, dihydroflavonols, flavonols, phlobaphenes, leucoanthocyanidins, proanthocyanidins, and anthocyanins. These subgroups are differentiated based on the oxidation state of the heterocyclic ring and the presence or absence of methyl or hydroxyl groups on the benzene ring. Currently, over 9000 different flavonoids have been identified and isolated from various plants. Plants contain a variety of flavonoids, including anthocyanins, which are primary pigments responsible for orange, red, purple, and blue hues. Aurones and chalcones are responsible for yellow coloring in plants. These flavonoids offer a broad range of colors to plants. Flavonoids also have antioxidant properties and function as phytoalexins, which protect plants against damage from biotic and abiotic stresses such as insect feeding, UV radiation, pathogen infection, and cold stress. Flavonoids also have the ability to scavenge reactive oxygen species (ROS), which can be damaging to plant cells [62]. Flavonoids are a class of phenolic chemicals with more than 6000 unique structural variations. The polyketide pathway, which produces polymeric C2 units, and the phenylpropanoid process, which develops the phenylpropanoid skeleton, are the two primary biosynthetic pathways in which flavonoids are generated in plants (C6–C3). The enzyme chalcone synthase creates the 2′-hydroxy chalcone scaffold from p-coumaroyl CoA and malonyl CoA as the initial step in the biosynthesis of flavonoids. Next, using different enzymatic processes, this scaffold is used to create a variety of flavonoids [63].

The initial step in flavonoid synthesis involves the conversion of phenylalanine into 4-coumaryl CoA, which is regulated by PAL, C4H, and 4CL enzymes. The second step involves the formation of dihydroxyflavonol from 4-coumaryl and 3 malonyl CoA, which is crucial in flavonoid metabolism and is regulated by CHS, CHI, and F3H enzymes. The third step involves the synthesis of total anthocyanins, and in the final stage, it is modified by glycosyltransferase and transported to the vacuole [64]. The results of the investigation into the *Cajanus platycarpus* flavonoid biosynthesis pathway genes in response to infection by *Helicoverpa armigera* demonstrated that certain genes, including CHS_1, CHS_3, DFR_3, DFR_5, F3′5′H_1, FLS_3, LAR_2, LDOX_1, and LDOX_2, exhibited a significant increase in expression levels, ranging from 4–11 fold. Additionally, moderate expression increases were observed in CHI_1, CHI_4, and CAR_1 genes, with up to a two-fold increase. Moreover, the study revealed that specific isoforms of these genes exhibited a response to insect herbivory. In *Cajanus platycarpus*, the accumulation of flavonoids was investigated in response to herbivory by *Helicoverpa armigera*. The results showed that t-ferulic acid had the highest accumulation, increasing by 5.7 times compared with other metabolites after 48 h of herbivory. The metabolite p-coumaric acid did not increase in concentration at 0 h without herbivory but showed higher accumulation after 24 h of herbivory. The accumulation of various flavonoids varied with the time of herbivory, with pelargonidin, myricetin, and naringenin showing higher accumulation compared with quercetin and kaempferol by 48 h. The study also found that the expression of flavonoid pathway genes and formation of metabolites were highly regulated in *C. platycarpus* during herbivory by *H. armigera* [65]. Tomato plants infected with tomato mosaic virus had higher total flavonoid content than AMF (arbuscular mycorrhizal fungi)-colonized plants compared with control plants. These findings suggest that the flavonoid biosynthesis pathway is activated in response to ToMV infection, and further enhanced by AMF colonization. The upregulation of specific genes involved in this pathway indicates a coordinated regulation of flavonoid biosynthesis in response to viral infection and AMF colonization. This may have important implications for the development of strategies to enhance plant resistance to viral infection by harnessing the potential of AMF to stimulate flavonoid biosynthesis [66].

#### 3.5.2. Coumarins

Coumarins are a group of secondary metabolites produced in various plant families, including Leguminosae, Poaceae, Apiaceae, and Rutaceae. They are derived from a phenolic compound produced during the shikimate pathway, and are commonly found in medicinal and aromatic plants. Coumarins are initially present in plant cells as O-coumaric acid glucoside and are converted into coumarin through enzymatic hydrolysis and lactonization during cell damage. They may play an essential role in plant defense against pathogens and insect herbivory. Coumarin has also shown promising results as an insecticide against *Myzus persicae* adult aphids. Coumarin showed high toxicity on aphids, whereas its high concentration did not cause toxicity on *Harmonia axyridis* ladybugs and *Eisenia foetida* adult earthworms, which reveals that coumarin selectively causes mortality to aphids [67]. Coumarins are generated in the phenylpropanoid pathway and are derived from 1,2-benzopyrones. There are several subclasses of coumarins, including simple coumarins, 7-oxygenated coumarins, phenyl coumarins, and pyranocoumarins. Phenyl coumarins are the most common coumarin compound and are produced during isoflavone metabolism. Simple coumarin, pyranocoumarins, and furanocoumarins, on the other hand, are all derived from the same pathway. Under biotic and abiotic stresses, *Arabidopsis* produces hydroxylated coumarins in the form of scopolin, which accumulate in stems and roots. Furanocoumarins, which can be of the linear or angular type, are effective phytoalexins and allelochemical compounds that are involved in plant–insect interactions. Plants with furanocoumarin accumulation possess an efficient biosynthetic pathway that can be induced by different types of stress [68]. Coumarins occur in plants either in their free state or as glycosides, and they have a polar structure. Due to their ability to absorb UV light, coumarins exhibit blue fluorescence characteristics. Some coumarins are photosensitive and can undergo structural changes upon exposure to natural light. Based on their chemical structure, coumarins are broadly classified into two groups: simple and complex coumarins. Simple coumarins, such as scopoletin, esculin, esculetin, umbelliferone, fraxetin, and sideretin, play various roles in the interaction of plants with abiotic and biotic stress. Complex coumarins are formed by adding heterocyclic compounds to the core structure of basic coumarins [69]. Coumarin derivatives, such as sulfonamide and dithioacetal, have been found to possess excellent anti-CMV (cucumber mosaic virus) activity. CMV is a plant virus that is widely distributed and can infect a variety of host plants, causing significant economic damage. Studies have shown that coumarin derivative C23 can enhance defense-related enzyme activity in tobacco, and induce the abscisic acid (ABA) pathway, resulting in improved defense responses against viral infections [70].

Plant-derived compounds involved in chemical defense are classified into two categories: phytoanticipins, which are continuously present in plant cells, and phytoalexins, which are produced in response to infections and not found in healthy plant tissues. Coumarins are among the plant compounds that accumulate during infections caused by different types of pathogens, such as bacteria, viruses, and fungi, in various plant species. Tanguy and Martin [71] reported that the inoculation of tobacco mosaic virus (TMV) on the leaves of the TMV-resistant *Nicotiana tabacum cv. Xanthi* cultivar led to the accumulation of coumarins in the developing necrotic lesions. Scopoletin has been produced in the *Hevea brasiliensis* rubber tree leaves due to infection caused by *Microcyclus ulei* fungus. The *Platanus occidentalis* tree exhibits resistance to the fungal leaf pathogen *Ceratocystis fimbriata p.* sp. *platani* by increasing the accumulation of coumarins, such as scopoletin and umbelliferone, at the site of infection. In vitro bioassays have shown that scopoletin is highly toxic to various fungi (*O. celmi*, *Batrytis cinerea*, *Cercospora nicotianae*, *Alternaria alternata*, and *the oomycete Phytophthora parasitica var. nicotianae*), bacteria (*Pseudomonas syringae*), and viruses (Tobacco mosaic virus). The ortho-hydroxylation of cinnamates, trans/cis isomerization of the side chain, and lactonization are used to create 2H-1-benzopyran-2-one, the structural core of coumarin, which is generated from cinnamic acid. In order to produce feruloyl CoA and take part in the production of coumarin scopoletin in the roots of Arabidopsis, the CCoAOMT1 gene, which encodes caffeoyl-CoA-O-methyltransferase 1, is essential [69]. Various microbe-associated molecular patterns (MAMPs) are crucial for activating defense mechanisms in plants. One such MAMP, flg22, was identified by flagellin-sensing 2 receptors and is known to enhance the production of scopoletin. The transcription factor MYB15 plays a key role in this process by regulating scopoletin synthesis. In the presence of MYB15, the synthesis of the feruloyl-CoA-6′-hydroxylase (F6′H1) enzyme is increased. Studies on Chinese wild grape have shown that the MYB15 promoter region is induced due to the immune response triggered by flg22. Both MYB15 and F6’H1 are essential for the production of coumarin scopoletin in plants. Another transcription factor, MYB72, is also considered a major factor in inducing scopoletin production [72].

#### 3.5.3. Lignin, Suberin, and Cutin

Lignin, an essential secondary metabolite produced through the phenylalanine/tyrosine metabolic pathway in plant cells, plays a fundamental role in plant growth and development. Its biosynthesis is a complex process consisting of three steps: (1) the production of lignin monomers, (2) their transportation, and (3) polymerization into lignin. The polymerization process involves the catalysis of three types of monolignols by peroxidase (POD) and laccase (LAC) in the secondary cell wall. Lignin, a major component of the cell wall that enhances its rigidity, aids in the transport of minerals through the vascular bundles and serves as a significant barrier against pests and pathogens. Additionally, lignin metabolic pathways are involved in the plant’s resistance and responses to environmental stressors [73]. Lignin, a complex polymer of phenylpropanoid compounds with extensive branching, serves several critical functions in plants, including mechanical support, facilitating water transport in the xylem, and providing defense against insects and pests. In response to pathogen attacks, an increase in lignification has been observed, which represents a defense mechanism to block pathogen invasion. In *Arabidopsis*, two genes were involved in cinnamoyl-CoA reductase (AtCCR1 and AtCCR2) being significantly expressed due to infection caused by *Xanthomonas campestris*. Chinese cabbage exhibited an accumulation of H_2_O_2_ and increased peroxidase activity in response to *Erwinia carotovora subsp. cortovora*, which resulted in the regulation of lignin production in plant cells. Ferulic acid, which acts as a precursor in lignin biosynthesis, was found to be present in the defense response against *Agrobacterium* in wheat. Lignin deposition was observed to increase in *Pinus nigra* when infected with *Sphaeropsis sapinea.* Lignification was also observed as a defense response in wheat against *Puccinia graminis*, with lignin-rich syringyl units being deposited [74].

Lignin deposition in plant cell walls contributes to increased mechanical strength and rigidity, which can improve resistance against lodging, or bending or breaking of the stem due to environmental stresses such as wind or heavy rain. Adding exogenous paclobutrazol to susceptible winter wheat cultivar enhanced lignin accumulation and increased the lignin biosynthesis enzyme’s activity, improving wheat tolerance against lodging. It was reported that analysis of the lignin metabolic pathway in *Fagopyrum esculentum Moench* shows that lignin production enzyme activities and lignin content play a critical role in disease resistance. The mineral element shows a fundamental role in plant lignin biosynthesis and resistance against disease; for example, the rice CAD gene expression can be enhanced by silicon which improves the accumulation of lignin and improves stalk strength which provides resistance. In contrast, the excessive presence of nitrogen fertilizer reduces lignin production and makes the crop susceptible to infections by weakening its stalk strength. Lignin is a complex polymer that provides structural support and rigidity to plant cell walls. Its accumulation is associated with the process of plant resistance to pests and insects. In rice plants, PAL (phenylalanine ammonia-lyase), C4H (cinnamate 4-hydroxylase), and PR9 (pathogenesis-related protein 9) genes are involved in lignin biosynthesis and are upregulated in response to insect infestation, leading to enhanced resistance to insects. Similarly, in *Chrysanthemum*, a transcription factor called CmMYB19 is induced by aphid penetration, which enhances the expression of genes involved in lignin biosynthesis and its accumulation. This results in reduced invasion of aphids in *Chrysanthemum*. Moreover, the rice toxin peptide LqhIT2 specifically targets insects and enhances lignin production mediated by jasmonate, leading to improved resistance to the leaf roller insect in rice plants. In maize, lignin biosynthesis and phenylpropanoid regulation might be facilitated by CCoAoMT and ZmCCoAoMT2, which were associated with multiple pathogen resistance traits [73].

Suberin is a hydrophobic polymer that is naturally deposited in the cell walls of specific plant tissues such as roots, tuber endodermis, and seed coats. It works together with related waxes to create a hydrophobic barrier that helps to regulate water and nutrient transport, gas exchange, and protect against pathogen invasion. Induction of suberin accumulation occurs due to an increase in the suberin in the suberized cell wall or the initiation of suberization among non-suberized cell walls. Deposition of suberin was induced by pathogen attack and wounds. It was known that plants synthesize suberin whenever they need to maintain a strong barrier. Suberin chemical composition involves a complex lipophilic polymer consisting of glycerol, aliphatics, and aromatic components associated with soluble waxes. The vital building blocks of suberin are interlinked as a polyester macromolecule, and a breaking ester reaction has been utilized for suberin depolymerization. Suberin and cutin are both complex polymers that contribute to the structural integrity of the plant cell wall and act as barriers to prevent water loss and protect against pathogens. However, while cutin is found primarily on the outer surface of the epidermal cell wall of the aerial parts of the plant, suberin is found in the root and stem tissues and is located in the inner layer of the cell wall, known as the Casparian strip, which forms a barrier between the cortex and the vascular tissue [75].

Suberin’s hydrophobic and resistant characteristics, which make it a strong deterrent against water loss and pathogen invasion, are provided by the polyphenolic domain. Moreover, the suberin polyphenolic domain has antibacterial substances that can stop some diseases from growing. The exact composition and structure of suberin can vary depending on the plant species, tissue type, and environmental conditions [76].

In the case of the highly-resistant rice germplasm Phule Radha, suberin accumulation was observed to be enhanced in the root tip, which is believed to contribute to its ability to efficiently resist RKN infections. In contrast, the susceptible rice variety PB1121 did not exhibit the same level of suberin accumulation and was more vulnerable to RKN infections. The expression levels of suberin biosynthesis genes play an important role in the defense of rice varieties against infection caused by *Meloidogyne graminicola*, also known as root-knot nematode (RKN). The Phule Radha variety showed increased accumulation of suberin and upregulation of suberin biosynthesis genes, particularly CYP86A1, CYP86B1, and MYB107, which may have contributed to its resistance against RKN. In contrast, PB1121 showed less expression of these genes and was more susceptible to RKN. These findings suggest that manipulating the expression of suberin biosynthesis genes may provide a strategy for developing resistant rice varieties against RKN infection [77].

The cuticle, an outer layer covering the aerial surface of plants, is composed of cutin and cuticular waxes produced by epidermal cells. This hydrophobic layer primarily functions to reduce non-stomatal water loss and is vital in plant–microbe interactions. The cuticle proper, containing a polymer of cutin with intracuticular waxes, is connected to the cell wall via a cuticular layer of polysaccharides and cutin. The outermost layer of the cuticle is composed of epicuticular wax, synthesized from wax crystal microstructures. Cutin is a three-dimensional polyester consisting of cross-esterified C16–C18 polyhydroxy and polyhydroxy fatty acids linked by a glycerol backbone. The cutin matrix provides the primary structural support for the cuticle [78]. Cutin enzymic polymerization mechanisms were characterized only a decade ago; 2-monoacylglycerols act as a cutin precursor and are produced in ER before it is transported to the apoplast. The cutin precursors are polymerized during transacylation reactions, catalyzed by the enzyme cutin synthase 1 (CUS 1), belonging to the GDSL family. The biosynthesis of cutin precursors involves multiple genes, including CUS2 and BDG1, which play a role in polymerization. In Arabidopsis, the transcription factor AtMYB16 has been shown to be essential for the biosynthesis of cutin precursors, as mutations in this gene resulted in altered expression of CYP86A4, a gene involved in the biosynthesis of hydroxy fatty acids. Another transcription factor, SHN1, has been identified as a regulator of cutin precursor biosynthesis genes [79]. The components of the cuticle and its breakdown products are observed to affect fungal infection and its development. Numerous studies have reported that both the hydrophobicity of the cuticle and the specific combination of wax components influence the process of pre-penetration by various pathogenic fungi. Investigation of *Medicago truncatula* showed that a mutation in the PALM1/RG1 (playmate-like pentafoliata 1/INHIBITOR OF RUST germ tube differentiation1) gene, which encodes a C_2_H_2_ zinc-finger-type transcription factor, can lead to reduced expression of MYB96 and CER4 genes in *A. thaliana*. This mutation has been found to confer resistance against rust fungi such as *Phakopsora pachyrhizi* and *Puccinia emaculata* [80].

#### 3.5.4. Tannins

Tannins, which are polyphenolic secondary metabolites, are abundantly present in the plant kingdom and play a crucial role in protecting plants from insect herbivory and pest attack. Based on variations in their chemical structure, tannins are categorized into three types: (1) hydrolyzable tannins, (2) phlorotannins, and (3) condensed tannins. Hydrolyzable tannins are composed of multiple glucose esters with gallic acid or ellagic acid, including gallotannins, glucogallin, ellagitannins, and their derivatives. These tannins can be degraded by acids, bases, and specific enzymes. Phlorotannins are formed by the polymerization of phloroglucinol monomer units and contain various compounds such as phlorethols, fucophloroethols, fucols, fuhalos, carmalols, and eckols. These tannins are synthesized in the acetate–malonate pathway and have a molecular weight ranging from 126–650 kDa. Condensed tannins are composed of polymer or oligomers of flavan-3-ol subunits linked by interflavan bonds of A-type or B-type and are categorized into prodelphinidin, prorobinetidins, procyanidins, and profistidins [81]. In plants, tannin serves a dual function; tannin protects plants against insects, pathogens, and herbivory and attracts insects to flowers, facilitating cross-pollination. Tannin precipitates digestive enzymes, which reduce food digestibility in animals and are classified as anti-nutritive compounds. In plant defense, tannins may exist in inactive forms called phytoanticipins or be induced into active states known as phytoalexins. It has been observed that the accumulation of proanthocyanidins, a type of condensed tannin, occurs during herbivory, wounding, and fungal attack in plants, indicating their critical role in plant stress response [82]. Tannins, which are water-soluble flavonoid polymers commonly found in vacuoles of various plants, exhibit toxicity against insects by binding to their digestive enzymes and salivary proteins such as trypsin and chymotrypsin, leading to protease inactivation. Herbivorous insects that consume high quantities of tannins present in plants may experience a decrease in weight gain and ultimately perish [83]. It is not yet known if the malignant lesions identified in the caterpillar *Orgyia leucostigma* midgut, particularly in the peritrophic envelopes, are directly related to the tannins or to oxidative stress [84].

Polyphenol tannins can either be tolerated by some insect herbivores or act as a toxin, making host organs less attractive to feed on. Tannins may serve as a disincentive, defensive compound, or even phagostimulants, depending on the environment. As galls induced by *Hartigiola annulipes* are rarely found damaged by herbivores, Pilichowski et al. assume that the tannin accumulation in *H. annulipes* galls acts as a deterrent for herbivores, thus promoting the survival of the gall inducer [85]. *Schlechtendalia chinensis* (Bell), a tiny insect of the Pemphigoid family, feeds on the adaxial surface of winged rachides, inducing the formation of large, single-chambered galls known as horned galls on *Rush chinensis.* The biosynthesis of different tannins in the gallnuts of *R. chinensis* provides a foundation for understanding how tannin production is maintained in response to interactions between host plants and insect herbivores. The identification of genes involved in tannin biosynthesis is not surprising since the production of tannins by plants is often associated with a defensive response against insect herbivores [80].

#### 3.5.5. Biphenyls and Dibenzofuran

The Rosaceae family is large in size (100 genera, 3000 species) and has commercial importance in fruit crops; however, little is known about its disease-resistance mechanisms. Among the rare discoveries was the first discovery of a phytoalexin, benzoic acid, which is formed in apple fruit after infection with *Nectria galligena*. Following that, biphenyl or dibenzofuran phytoalexins have been found and examined in six Rosaceae species. These phytoalexins have been identified in the sapwood of Cotoneaster, Malus, and Pyrus, as well as Eriobotrya, Photinia, and Rhaphiolepis leaves. The sapwood of 29 species of Maloideae produced large amounts of a diverse range of mostly novel phytoalexins. The phytoalexins were five biphenyls l–5 and fourteen dibenzofurans compounds. Biphenyls and dibenzofurans are produced, and their formation within the Rosaceae appears to be taxonomically relevant. There are two exceptions to the rule that plants in the same genus produce the same type of phytoalexin. The phytoalexin reaction of diverse tissues in *Eriobotrya japonica* is remarkable. Thus, the cortex creates biphenyl aucuparin, but the leaf produces dibenzofuran [86]. Another example is found within the genus Sorbus, where different species produce variable amounts of phytoalexins. Interestingly, the absence of any plant tissue investigated thus far that produces both biphenyls and dibenzofurans suggests that, despite their close relationship, these two groups of phytoalexins are synthesized via parallel rather than sequential routes.

Aronia produces biphenyls, which may be a useful indicator of the genus’ affinity for Sorbus. As a result, a phytogeographical hypothesis that Crataegus (North America) diverted to Aronia and Malacomeles (Central America) and Hesperomeles (South America) while migrating southward has been proposed. An examination of a few naturally damaged wood tissues yielded more intriguing results, though thorough structural identification is still pending. The UV absorption spectra of the semi-purified, most significant fungitoxins in the sapwood of *S. intermedia* (subgenus Aria), which were not detectable in healthy tissue, resemble those of dibenzofuran. *S. torminalis* (subgenus Torminaria) developed two phytoalexins with UV spectra that could not be assigned to either biphenyl or dibenzofuran. Their chemical identities are currently being researched [87].

**Table 6:** Phenolic compound response to biotic stress.

Phenolic Compound	Plant Name	Type of Stress	Response of Phenolic Acids to Stress	Reference
Flavonoid	*Cajanus platycarpus*	Herbivory by *Helicoverpa armigera*	Increase in flavonoid content	[65]
Scopoletin	*Hevea brasiliensis*	Fungal infection by *Microcyclus ulei*	Increase in scopoletin level	[69]
Coumarin, scopoletin	*Plantanus occidentalis*	Fungal infection by *Ceratocystis fimbriata* and *Ceratocystis platani*	Increase in coumarin scopoletin level	[69]
Lignin	*Pinus nigra*	Blight disease by *Sphaeropsis sapinea*	Lignification increases	[74]
Lignin	*Triticum* spps.	Stem rust by *Puccinia graminis*	Increase in lignin level	[74]
Caffeic acid	*Zea mays*	Leaf blight by *Glomerella Graminicola* or *Cochliobolus heterostrophus*	Increase in two phenolic caffeic acid esters	[88]
Chlorogenic acid, cinnamic acid	*Vigna radiata*	Infection by *Meloidogyne javanica* (root-knot nematode)	Increase in chlorogenic acid and trans cinnamic acid	[89]
Chlorogenic acid, catechin	*Nicotiana attenuata*	Infection by *Trichobaris mucorea*	Increase in chlorogenic acid and catechin	[90]
Cinnamic acid, naringin, and rutin	*Beta vulgaris*	Infection by *Canavalia ensiformis*	Increase in cinnamic acid, naringin and rutin	[91]
Pterostillbene and resveratrol, piceide	*Vitis vinifera*	Downy mildew by *Botrytis cinerea*	Increase in pterostillbene and resveratrol, piceide	[92]
Aucuparin,2′-Hydroxyaucuparin 2′-Methoxyaucuparin	*Aronia arbutifolia* (L.) *Elliott*	Due to fungal inoculation or natural infection	Aucuparin,2′-Hydroxyaucuparin 2′-Methoxyaucuparin (biphenyl induction)	[87]
Aucuparin 2′-Hydroxyaucuparin 2′-Methoxyaucuparin 4′-Methoxyaucuparin Isoaucuparin	*Sorbus aucuparia* L.	Due to fungal inoculation or natural infection	Aucuparin 2′-Hydroxyaucuparin 2′-Methoxyaucuparin 4′-Methoxyaucuparin Isoaucuparin (biphenyl induction)	[87]
Xanthone	*Hypericum perforatum (HP*) suspension cultures	Elicitation by *Agrobacterium tumefaciens* cultivation	Xanthone biosynthesis in HP cells	[93]
Xanthone	Hairy roots of *Gentiana dinarica*	Under biotic stress (chitosan and yeast extract)	Accumulation of xanthone	[94]

## 4. Storage of Secondary Metabolites

The buildup of secondary metabolites, which serve as a natural defense of plant cells, could be deleterious to the tissues of the plants. Plants are evolved with specialized structures for storing such harmful metabolites. Water soluble metabolites were found to be stored in vacuoles while lipophilic molecules were stored either in latex, resin ducts, trichomes, glandular hairs, etc. [15]. The cell walls enclosing storage cavities frequently possess thick lignified or suberized cell walls in order to prevent their diffusion away from storage structures since storage structures frequently include typical volatile compounds such as monoterpenes [95]. In Table 7, a comprehensive list of plant storage organs is provided along with the different types of metabolites associated with each organ.

### 4.1. Latex

The specialized cells (or row of cells) known as laticifers produce and store latex. The chemical makeup of the latex formed varies greatly, often appearing white rather than densely milky, and carrying a wide range of dissolved solutes and macromolecules as well as suspended colloids [96]. The performance of herbivores can be decreased by poisonous metabolites in latex while the adhesive properties of latex allow for the capture of whole insects or the binding of their mouthparts together [97]. Turgor pressure is generated inside laticifers as a consequence of latex storage. When a plant is wounded, latex flows profusely in reaction to pre-existing turgor pressure since the laticiferous structure provides an uninterrupted cellular gap along the plant body. Natural opium poppy (*Papaver somniferum*), papain (peptidase) from papaya trees (*Carica papaya*), cardiac glycosides of milkweeds (*Asclepias* spp.), and phenolic glucosides from hemp (*Cannabis sativa*) are just a few of the beneficial bioproducts that come from the metabolites that arise in latex [98].

In *Euphorbia lathyris*, laticifers are a cellular specialization for a crucial coping strategy to ward off arthropod predators with various eating patterns, such as *Spodoptera exigua* and *Tetranychus urticae* [98]. The polyisoprenes that delay the contact and growth of pathogens and terpenes, such as 24-methylenecycloartanol with antifungal properties, are significant features of the latex of *Euphorbia* species [99]. Likely, the latex of Apocynaceae members is abundant with phenolics, terpenoids, neutral lipids, alkaloids, mucilage, resin acids, pectin, carboxylated polysaccharides, hydrophilic and lipophilic compounds, and proteins that inhibit herbivory and discourage microorganisms [100]. The phenolic inositol esters (PIEs), β-D-glucopyranosyl esters, sesquiterpene and triterpene acetates (TritAcs), and lactone taraxinic acid are the major constituents of the latex of *Taraxacum officinale* agg., which shield the plant against herbivore attacks [97].

### 4.2. Trichomes

Oftentimes, glandular trichomes (GTs) are referred to as “the first defense line” of plants against abiotic and biotic threats because of the variety of natural compounds present in trichomes, which predominantly function as defense molecules [101]. Trichomes can be non-glandular or glandular, multicellular or unicellular, and have grown on almost all plant parts of terrestrial plants, including, stems, leaves, and even flowers and fruits. They frequently act as physical defenses against fungal infection and insect invasion [102]. Glandular trichomes also provide storage spaces for VOCs, fatty acid derivatives, and terpenes which could potentially draw natural enemies that indirectly safeguard host plants. [103]. When immature shoots are extremely susceptible to leaf- and stem-feeding insects and before the resin duct system typical of conifers has fully matured, spruce glandular trichomes may offer an early, terpene-based chemical defense strategy [104].

The tips of the trichomes are found to be metabolically active regions and contain several alkaloids, terpenoids, and phenolics [105]. The phenolic chemicals that are housed can be released when the glandular trichomes are harmed by insects. Polyphenol oxidase then converts the released phenolic compounds to quinines, rendering insects unable to feed since they are “glued” to the surface of the leaf. The antioxidant qualities of such molecules also shield plants against microbial attacks by limiting the generation of unwanted reactive oxygen [106].

Apart from these, secondary metabolites can also be found in resin ducts, floral nectaries, oil glands, etc. In coniferous plants, the defensive volatile compounds and terpenoids such as pinenes, limonene, terpinolene, caryophyllene, and resin acids are mainly stored in resin ducts [107]. Following an herbivore attack, this resin will begin to release. It can either poison the attackers or act as glue to hold the herbivores’ mouthparts together. Extrafloral nectaries are also considered as storage organs for secondary metabolites. Cassava, tapioca, passiflora, cotton, cashew nut trees, and several members of the Rosaceae have been reported to emit secondary metabolites from extra floral nectaries that aid in luring predators of various pest species [108,109].

**Table 7:** List of different metabolites in various plant storage organs.

Metabolite	Plant	Storage Structure	Storage Tissue	Property	Reference
Artemisinin	*Artemisia annua*	Glandular trichome	Epidermal extensions	Anti-malarial	[110]
Gossypol and related compounds	*Gossypium hirsutism*	Trichome	Epidermal extensions	Antifungal	[111]
Pyrethrin	*Tanacetum cinerariifolium*	Trichome	Epidermal extensions	Insecticide	[112]
Essential oil	*Ocimum species*	Glandular trichome	Epidermal extensions	Antimicrobial	[113]
Alkaloids	*Robinia viscosa var. hartwigii*	Glandular trichome	Epidermal extensions	Antimicrobial	[114]
Phenolic compounds	*Millingtonia hortensis*	Glandular trichome	Epidermal extensions	Herbivore and insect resistance	[115]
Phylloplanins	*Nicotiana tabacum*	Glandular trichome	Epidermal extensions	Antifungal	[116]
Acyl sugars	*Solanum lycopersicum*	Glandular trichome	Epidermal extensions	Insect and mite resistance	[117]
Acyl sugars	*Capsicum annuum*	Glandular trichome	Epidermal extensions	Pathogen resistance	[118]
Terpene compounds	*Picea abies*	Resin ducts	Epithelial parenchyma cells	Insect and pathogen resistance	[107]
Phenolic and terpenoid compounds	*Rivea ornata* (Roxb.)	Extrafloral nectaries	Epithelial parenchyma cells	Attracts predators of pests	[108]
β-D-glucopyranosyl ester	*Taraxacum officinale*	Latex	Laticifers	Anti-herbivore	[97]
Polyisoprenes	*Euphorbia* spp.	Latex	Laticifers	Antifungal	[99]
Alkaloids	Apocynaceae members	Latex	Laticifers	Anti-herbivore	[100]

## 5. Enzymatic Regulation of Secondary Metabolites under Biotic Stress

Due to their abundance of metabolites, plants are well known for being adept chemists. Primary metabolites and secondary metabolites are two categories into which plant metabolites are frequently subdivided. Primary metabolites are ubiquitous in all plants and are essential for their growth and development. Secondary metabolites are unique to plants and are involved in a variety of biological and physiological activities, including defense and adaptation to harsh situations. Plant secondary metabolites (PSMs) can be divided into three groups based on their main structural characteristics: phenolics, terpenoids/isoprenoids, and chemical compounds that include nitrogen. Similar to an animal’s adaptive immune system, some metabolites in plants play a critical role in defending them against a variety of biotic and abiotic threats. At various points in the development of a complete plant defense system, PSMs serve as antioxidants or messenger molecules. They are also crucial signaling pathway starters that begin the production of genes associated with defense and the accumulation of other metabolites. Alkaloids are a broad class of heterocyclic chemical molecules with nitrogen that are divided into five divisions based on their chemical makeup and precursor amino acids: terpenoid indole alkaloids (TIAs), tropane alkaloids, benzylisoquinoline alkaloids (BIAs), pyrrolizidine alkaloids, and purine alkaloids. The exploration of the intricacy of alkaloid biosynthesis uses TIA and BIA as examples. The Ranunculales order of plants frequently contains specialized metabolites known as benzylisoquinoline alkaloids, which are structurally unique from other plant metabolites. Dopamine and 4-hydroxyphenylacetaldehyde, a tyrosine derivative, produce the fundamental precursor trihydroxylated alkaloid, which serves as the starting point for the biosynthesis of BIAs [119].

### 5.1. Peroxidase

Another family of heme-containing proteins, peroxidases exhibit a wide range of structural variation and preferentially oxidize aromatic electron donors such as guaiacol and pyrogallol at the expense of H_2_O_2_. Vacuole or cell wall class III peroxidase, which catalyzes different substrate oxidation and plays a crucial role in plant defense systems and numerous biosynthetic pathways under stress conditions, are typically present in the apoplast. Depletion of hydrogen peroxide is necessary for lignin biosynthesis and defense against biotic stressors in several cell areas, including cell walls, vacuoles, and extracellular space [120]. Glycoproteins have been synthesized by the endoplasmic reticulum and golgi apparatus as peroxidase, which caused their secretion into vacuoles or the extracellular area. Plant peroxidase (POX) is a single polypeptide with 300–350 amino acid residues and a mass of 33–55 kDa found in most plants. Guaiacol, a POX enzyme, is a common reducing substrate for its H_2_O_2_-dependent oxidation by guaiacol peroxidase (GPOX) [121].

The significance of POXs in defense against pathogen infection has been demonstrated by several studies. The resistance of tobacco to wildfire disease and POX activity were found to be positively correlated in tobacco. A strain of *Xanthomonas oryzae* pv. oryzae quickly increased the amount of POX isoenzyme in rice plants when it infected the plants. POXs play the following important roles in plant defense: (1) strengthening the cell wall into a physical barrier consisting of suberin, lignin, hydroxyproline-rich glycoprotein, and feruloylated polysaccharides, (2) improving reactive oxygen species generation via antibacterial agents and signal mediators, and (3) increasing the synthesis of phytoalexin. Increased levels of POX mRNA and protein activity have been observed in plants in response to a variety of stimuli such as pathogen infection, oxidative stress, and wounding. In wounded plant tissues, a group of defense-related genes known as wound-inducible POX genes are activated, resulting in the production of main-type enzymes and PR proteins involved in phenylpropanol synthesis. Low-molecular-weight substances such as ABA, jasmonate, and ethylene have an effect on these genes, which are expressed as a result of signal transduction pathways. When the plant was injured, the concentration of POX genes, which were identified from tobacco plant transcripts, increased consistently. Several studies have demonstrated that defense-related signal chemicals such as ABA, jasmonic acid (JA), and salicylic acid (SA) enhance the mRNA level of the pathogen-inducible POX gene. For instance, ABA therapy of tomato, JA treatment of rice, and SA treatment of parsley and cucumber. In contrast, a previous study discovered that the tobacco POX gene, which is activated in leaves infected with the tobacco mosaic virus during N-gene-dependent hypersensitive reactions, did not respond to plant defense signaling molecules [122].

### 5.2. Lipoxygenase

Lipoxygenase (LOX), discovered by Bohn and Haas in 1928 as a carotene oxidation enzyme, is a widely distributed oxidoreductase enzyme found in both plants and animals. LOX is produced by a variety of dioxygenase redox enzymes with molecular weights ranging from 90 to 110 KDa that contain non-heme iron, non-sulfur iron, and manganese. LOX is divided into two domains: the N-terminus and the C-terminus [123].

Lipoxygenase (LOX) is a well-known enzyme in plants that plays a variety of roles in fruit ripening, tuber growth, seed germination and, most notably, plant defense mechanisms. Polyunsaturated fatty acids (PUFAs) are oxidized by LOX after insect or pathogen assaults. Plant LOXs can be divided into two major groups, called 9-LOX and 13-LOX, which are characterized by the exact location at which linoleic acid is oxygenated in each group [124]. Plants induce metabolite production with preventive features during pathogen attack. The main oxylipins implicated in LOX pathways, which are generated by 13-LOX and 9-LOX, respectively, include 10-OPDA, 12-OPDA isomer, and 10-oxo-11-phytodienoic acid (10-OPEA), which are vital for plant defense against chewing and biting herbivores. Moreover, it has been found that the 9-LOX pathway is crucial for defense against pathogen assaults such as those brought on by *Pseudomonas syrigae*. The “death acids” produced by the *Zea mays* LOX pathway, collectively known as 10-OPEA, 12-OPEA, and 14-C cyclopentenones, are crucial in boosting the production of jasmonic acid after *Cochliobolus heterostrophus* infection. These infections promote cytotoxicity, which causes cell death, as well as the activity of genes involved in defense and phytoalexins. Infection caused by *Fusarium verticillioides* and *Cochliobolus carbonum* activated two segmentally 9-LOX duplication genes, ZmLOX4 and ZmLOX5, of *Zea mays* to carry out resistance mechanism [123]. 

According to research on LOXs from diverse plants, Arabidopsis has six LOXs (AtLOX1-AtLOX6) in its genome. AtLOX1 regulation was enhanced during attack on leaves and synthesis of stress-related hormones [124]. In *Arabidopsis thaliana*, it was observed that the 9-LOX pathway modifies plant defense, oxidative stress, and lipid peroxidation. Pathogen attack in pepper leaves improves the expression of CaLOX1 and the 9-LOX gene; moreover, in *Arabidopsis thaliana*, enhanced expression of CaLOX1 provides resistance towards *Alternaria brassicicola*, *Pseudomonas syringae*, and *Hyaloperonospora*. 9-LOX oxylipins provide defense against the initial infection of *Phytophthora infestans* in potatoes. In *Zea mays,* feeding larvae *Spodoptera exigua* improve the 9-LOX expression to a greater level than 13-LOX or 3-LOX by invasion of *Aspergillus flavus* and *Fusarium verticillioids*. *Magnaporthe grioea* infection in rice leaves enhanced LOX3 activity [123]. A study of the tobacco genome in the experimental setup showed that among 12,13-LOX and 19,9-LOX genes, six and twelve genes were expressed effectively. In both typical and latent *Pectobacterium atrosepticum* infections, two genes exhibited a ten-fold increase in expression levels, with higher expression during typical infection, and only one gene was expressed during latent infection. This indicates that the 9-LOX and 13-LOX pathways of the lipoxygenase cascade are expressed during infection [125].

### 5.3. Polyphenol Oxidase

Polyphenol oxidases (PPOs) consist of structurally similar catechol oxidases (COs) and enzyme tyrosinases (TYRs). Polyphenol oxidases are a type of metalloenzyme that consist of a type III copper center. The polyphenol oxidase family comprises three types of enzyme tyrosinases (TYRs), aurone synthases (AUSs), and catechol oxidases (COs). The PPOs of tomato plants are engaged in plant defense mechanisms as it was observed that PPO suppression increases the susceptibility of plants to diseases, whereas overexpression of PPOs improves plant resistance against microorganisms such as *Pseudomonas syringae.* In addition, PPO activity in tomato plants provides resistance against herbivore insects such as common cutworm (*Spodoptera litura*) [126]. Polyphenol oxidase (PPO) has been shown to play a role in plant defense against insect herbivores. Felton et al. found that there is an inverse correlation between PPO levels and the growth of the *Heliothis zea* caterpillar on tomato plants, suggesting that PPO may have an anti-nutritive effect on the caterpillar. Furthermore, systemin and MeJA have been shown to induce PPO activity and mRNA levels in tomato plants, supporting their role in herbivore defense. Although the PPO gene family is known to be highly conserved, different members may exhibit varying expression patterns in response to different stimuli, highlighting the complexities of their roles in plant defense. In tobacco, NtPPO1, NtPPO2, and NtPPO3 genes have different responses to MeJA, ABA, wounding stress, and whitefly infestation. An inducible defensive protein known as polyphenol oxidase aids in defending *Camellia sinensis* (Tea) against larval pests, particularly *Ectropis grisescens*. It has been discovered that the signaling of jasmonic acid is connected to this defense mechanism [127].

When nematodes migrate inside the plant, the root system induces free phenol production in root cells which react with oxidative enzymes present in plants such as PPO and POD (peroxidase) that form quinone toxicity. By boosting the production of phenolic compounds and the activity of enzymes such as PPO and POD, plants engage their defensive mechanism against nematode-caused root lesions. These substances and enzymes prevent nematodes from feeding and reproducing, acting as a chemical and physical barrier. Studies reported that banana cultivar and *Pratylenchus coffeae* interaction in non-inoculated and inoculated resistance treatment showed an increase in the level of defense enzyme PPO. Additionally, in tomato, the amount of PPO and phenolic compound chlorogenic acid increased to provide protection against *Pratylenchus penentrans*, indicating the induction of the defense response [128]. Polyphenol oxidase presence strengthens plant defense; when pathogens damage plant membranes, chlorogenic acid produced from phenols creates an unfavorable environment for the pathogen, restricting its spread in plants. PPO activity in bananas was shown to be elevated during bunchy top infection in banana cultivars. Moreover, PPO combined with phenol was shown to cause defense activity against pathogens in plants [129]. Furthermore, PPO1 gene regulation during stress reactions in mulberry plants is aided by MnMYB3R1 binding to a specific area in the promoter region. PPO activity can be markedly induced in several species, including radish seedlings, ajowan, jujube, *Brassica juncea*, eggplant, kiwifruit, and *Citrus unshiu*, by a variety of stressors as well as phytohormones. The antisense transformation of the potato PPO gene and its introduction into transgenic tobacco led to a decreased responsiveness of the NtPPO gene and PPO activity in response to herbivory and injury [127].

### 5.4. Glycosyltransferases

Glycosyltransferases (GTs) belong to the multigene superfamily in plants that are capable of transferring activated single or multiple sugars among various plant molecules, which cause glycosylation of plant compounds [130]. The glycosylation process, which is aided by an enzyme class known as glycosyltransferases, results in the formation of glycans, and the CAZy database finds 105 genes implicated in this process. According to research, the majority of GTs participate in the post-synthesis glycosylation of lipids and proteins in the Golgi apparatus. It has been discovered that the mechanism of protein N-glycosylation, which is likewise made possible by GTs, involves the systematic organization of these enzymes in protein complexes that are dispersed among various Golgi cisternae to carry out particular tasks [131]. The alteration of glycosylation, or the joining of sugar molecules to other molecules, broadens the variety of secondary metabolites found in plants. The stability, bioavailability, and solubility of these molecules are changed by this alteration. Glycosyltransferases (GTs), which are members of family 1 of the carbohydrate active enzyme (CAZY) database and employ uridine diphosphate (UDP) as a sugar source, enable the glycosylation of plant secondary metabolites. GTs implicated in glycosylation of plant secondary metabolites exhibit a conserved motif of 40 amino acids towards the C-terminus, known as the plant secondary product glycosyltransferases (PSPG) box. The PSPG box in GTs is an important characteristic that renders them soluble enzymes, which is useful for expressing these enzymes in heterologous hosts [132].

Glycosyltransferases (GTs) are generally grouped based on amino acid sequence similarity, and 114 GT families have been found so far. By transferring the glycosyl group of UDP sugar to a hydroxyl group of acceptor molecules, GT1s from family 1, also known as uridine 5′-diphosphate (UDP) GTs or UGTs, catalyze the glycosylation of flavonoids in plants. There are 120 and 244 UGTs in *Arabidopsis thaliana* and *Oryza sativa*, respectively. The primary glycosylation residues are the flavonoid sugar’s 3-, 5-, 7-, and 4′-OH acceptor sites [133]. In Arabidopsis, three homologous enzymes from the UGT78D family catalyze the initial steps of 3-O-glycosylation on flavonols. Although kaempferol and quercetin are the same isorhamnetin aglycone substrate for all three of these enzymes, their specificity for the sugar donor distinguishes them from one another [134]. Several data indicate that UGTs contribute significantly to plant disease resistance. For instance, the conjugation of hydroxycoumarins led to the induction of Togt1 and Togt2 in tobacco after a hypersensitive response, which demonstrated excellent efficiency. Transgenic tobacco plants were found to become more vulnerable to tobacco mosaic virus when TOGT was silenced in them (TMV). Upon TMV and *Pseudomonas syringae* inoculation, respectively, UGTs such as CaUGT1 in *Capsicum annuum* and UGT73B3 and UGT73B5 in *Arabidopsis thaliana* were induced. Several investigations using mutant versions of the UGTs ugt73b3 and ugt73b5 revealed that both UGTs played a key role in controlling the redox reaction during Arabidopsis resistance to bacterial infection [135].

## 6. Role of Secondary Metabolites in Biotic Stress Tolerance

### 6.1. Secondary Metabolites in Indirect Defense Mechanism

#### 6.1.1. Secondary Metabolites in Allelopathy

Plant allelopathy is a vital part of plant defense that entails the discharge of a number of low-molecular-weight secondary metabolites from plants that are believed to influence their surroundings by limiting the reproduction or development of neighboring plants [136]. It has been demonstrated that certain weed species, such as *Chenopodium album*, *Avena fatua*, *Portulaca oleracea*, *Lolium rigidum*, and *Bromus japonicus*, are inhibited by allelochemicals such as flavonoids and phenolic acids, phenoxazinones and benzoxazinones, from different wheat genotypes [137]. Similarly, cuminaldehyde from *Cuminum cyminum* L. was found to hinder the development of various monocot and dicot plants [138]. Avenicin is one of the vital allelochemicals with powerful activity against plant pathogenic organisms, produced by oats [136]. The growth and availability of nitrogen for the invasive community are impacted because secondary metabolites (procyanidins) discharged by *Fallopia* spp. have recently been demonstrated to impede the bioavailability of nitrogen in the rhizosphere of invader plants by limiting the microbial denitrification [136,139,140].

#### 6.1.2. Secondary Metabolites to Draw Predator Enemies

Plants use volatile organic molecules as an induced indirect defense mechanism to lure natural predators of herbivores [141,142]. HIPV or herbivore-induced plant volatiles primarily consist of terpenoids, phenylpropanoids/benzenoids, and fatty acid and amino acid derivatives [143]. Microplitis mediator, an insect predator, which is drawn to the volatile chemicals from cotton plants infected with *Agrotis segetum*, is an effective indirect defense mechanism [144]. Likewise, aphid- and caterpillar-infected cucumber and potato plants release HIPVs as a cue for ants [145]. Similarly, the volatiles released by *Arabidopsis* infested with aphids and caterpillars have been shown to attract parasitoids, such as *Diadegma semiclausum*, a significant opponent of herbivorous insects [146]. It was also reported that *P. brassicae* plants harmed by larvae and nymphs of other non-hosts produce volatiles that entice Trichogramma wasps [147]. Recent studies reveal that insectivorous birds also depend on herbivore-induced plant volatiles as a hint to seek their prey [148,149,150,151]. Apart from these, plants’ extra floral nectaries also contain certain secondary metabolites to attract ants, which help them to be less attacked by herbivores [152].

#### 6.1.3. Secondary Metabolites as Cellular Barriers

Cutin, suberin, and lignin are examples of secondary metabolites that significantly contribute to the wound-healing process, which largely guards against pathogen infection and maintains post-injury survival [78]. Two late immunological responses that strengthen plant cell walls are the accumulation of lignin and callose [153]. Lignin accumulation can be considered as a barricade against the transmission of pathogens that reduces the accessibility of fungal enzymes and toxins into plant cell walls [73]. Apart from lignins, suberins also play a significant role in the endodermal barrier system, including Casparian strips in plants to defend root nematodes [154]. The deteriorated endodermis by nematode invasion has been found to be defeated by depositing suberin in the periderm in *Arabidopsis* [155]. Comparable to this, the presence of the specific antimicrobial phenolic compound xanthone in the endodermis and exodermis of *Hypericum perforatum* roots similarly shields the plants from soil-born microorganisms [93,156]. Apart from cellular barriers, conjugation of suberins with hydrocinnamic acid derivatives (HCAAs) provides antimicrobial and antifungal properties. HCAAs work to strengthen cell walls and slow down cell wall deterioration to fend off pathogen infection [153].

#### 6.1.4. Secondary Metabolites as Regulators of Plant Defense

It has now been established that at least five kinds of secondary metabolites, such as benzoxazinoids, terpenes, glucosinolates, green-leaf volatiles, and aromatics, can potentially regulate in-plant defense against biotic stress [14,22]. According to Clay et al. (2009), plant glucosinolates along with the genes PEN2 and PEN3 play an important role in the production of callose plugs in the cell walls of pathogen attack to restrict further pathogen entry [157]. The activation of the PR1 gene and callose deposition was also boosted by the exogenous administration of coumaroyltyramine and coumaroyltryptamine in *Arabidopsis thaliana* [158]. Low amounts of ferulic acid considerably regulated stomatal opening, stomatal opening rate, stomatal width, and stomatal length because stomatal closure can prevent bacterial invasion and decrease bacterial infection [159]. In support of this, the secondary metabolites campesterol, sinigrin, sitosterol, and quercetin were found to control stomatal closure in *Arabidopsis thaliana* and *B. napus* [160]. The significance of the glucosinolate–myrosinase system in stomatal closure was also reported by Ahuja et al. (2021) [161].

#### 6.1.5. Secondary Metabolites to Deter Herbivores and Pathogens

##### Feeding Deterrents

An antifeedant is a substance that prevents insects from eating but does not really kill them. The insect hangs in the plant parts until it starves to death. Antifeedants are promising, ecologically friendly pest control agents for plants and stored goods. As the underlying mechanisms driving antifeedant activity are still unclear, it is only conceivable that antifeedants reduce or stop insect feeding through processes involving chemosensory-based food aversion [162].

According to Lin et al. (2021), 7-deacetyl gedunin, azadirone, gedunin, salannin, salannol, methyl angolensate, azadiradione, and azadirachtin of the Meliaceae family have been found to be potential feeding deterrents for various insects [163], particularly lepidopterans [164]. Similarly, the antifeeding properties of various limonoids such as dumnin, dumsenin [165], musidunin, musiduol [166], umketol, zumsenin, and zumsenol [167] from *Croton jatrophoides* against a wide variety of feeding insects have also been reported. Pyrethrins also offer similar feeding deterrence against various sucking insects [168].

##### Oviposition Deterrents

Herbivorous insects lay eggs on plants, and as the eggs hatch, the larvae that will eventually eat them pose a hazard [169]. Oviposition deterrents are the metabolites that stop insects from laying eggs [170]. Coumarin and rutin have been found to be effective oviposition deterrents in cabbage. The efficiency of ginsenosides present in the *Panax ginseng* plants on the feeding behavior and oviposition-deterring activity of *Pieris rapae* has been established [171]. Plant secondary metabolites such as azadirachtin and eucalyptol are also potent oviposition deterrents of lepidopteran insects [172]. According to Lin et al. (2021), acyl sugars act as oviposition deterrents against various thrips, such as tobacco thrips, *Frankliniella fusca* (Hinds), and western flower thrips [173], and *Frankliniella occidentalis* in Solanaceae members [174].

#### 6.1.6. As Apostematic Signals

Numerous herbivores have been suggested to be repelled by the red color of anthocyanin leaves [175]. The optical properties of anthocyanins may serve as a visual cue to potential herbivores that there is a considerable metabolic investment in toxic or unpleasant chemicals. Anthocyanins have also been linked to the mimicking of defensive structures, the dismantling of insect crypsis, and the concealment of plant components against their backgrounds [176]. According to Chen et al. [177], the red color of spring leaves caused by the anthocyanin build up is a common defense mechanism to deter lepidopteran and coleopteran insects. The findings of Menzies et al. [178] also support the importance of red pigments in herbivore deterrence. Anthocyanins exhibit antifungal properties too [179].

### 6.2. Secondary Metabolites in Direct Defense

#### 6.2.1. Phytotoxins

To defend themselves against numerous threats such as bacteria, fungi, insects, and predators, plants naturally produce phytochemicals or secondary metabolites called phytotoxins. According to Akama Friday [180], these phytotoxins are capable of destroying or killing herbivores since they have fatal neurotoxic and cytotoxic effects. Following herbivore attack, cyanogenic glycosides such as linamarin, lotaustralin, and amygdalin of cassava, dhurrin of sorghum, and taxiphyllin of bamboo shoots are released and react with β-glucosidase to produce sugars and a cyanohydrin that spontaneously decomposes to HCN, a strong neurotoxin which impairs cellular respiration [181]. Similarly, alkaloids such as caffeine (coffee, tea, and cocoa), nicotine (tobacco), morphine, and codeine (opium poppy) are also potent plant-derived toxins that cause metabolic as well as enzymatic alterations in herbivores [182].

#### 6.2.2. Phytoalexins and Phytoanticipins

**Figure 2:**
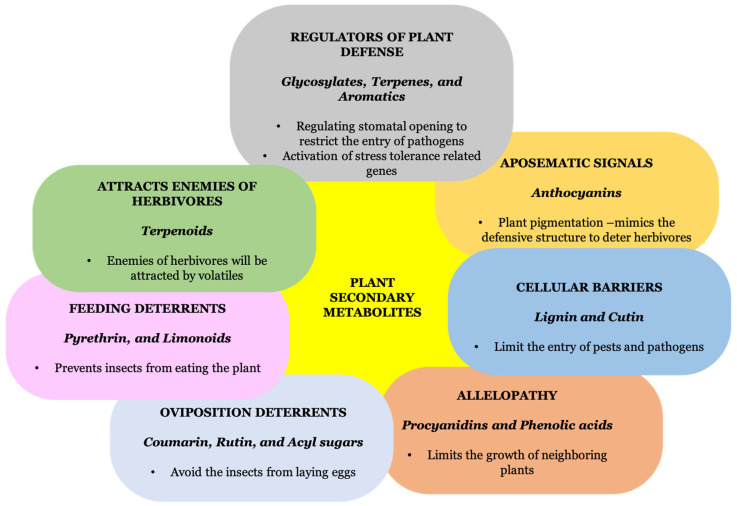
Role of plant secondary metabolites in biotic stress defense.

Low-molecular-weight antimicrobial compounds known as phytoalexins are induced by biotic stress or infection as a component of their active defense mechanisms in plants [183,184]. Phytoalexins are specific to plant families, such as isoflavonoids in most legumes and terpenoids in the Solanaceae family [185], glucosinolates in cruciferae, Phaseollin in beans, pisatin in peas, glyceollin in soybeans, alfalfa, and clover, rishitin in potatoes, gossypol in cotton, and capsidiol in pepper are some of the phytoalexins that have been more thoroughly examined [186]. The cornerstone of biotic stress tolerance is phytoanticipins, which are plant antibiotics that are present in tissue before infection [187]. Saponins are considered phytoanticipins in many plants. Avenacin in oats and α-tomatine in tomato are some of the well-studied phytoanticipins [188]. Figure 2 illustrates the pivotal role of plant secondary metabolites in biotic stress defense.

## 7. Signaling Mechanism under Biotic Stress

Numerous modifications were initiated at the genomic level once the stress signals were established in the plant cell system. Various signaling pathways were activated by specific promoters in order to combat the stress induced in the system. When biotic-stress-inducing signal molecules such as MAMPs (microbe-associated molecular patterns) or PAMPs (pathogen-associated molecular patterns) bind to PRRs (pattern recognition receptors), PTI (PAMPs-triggered immunity) or MTI (MAMPs-triggered immunity) sets in by consecutive calcium influx and reactive oxygen species generation [189]. Salicylic acid, a significant plant hormone, plays a vital role in biotic stress tolerance. Reports show that it is crucial in controlling ROS levels and ultimately helps in avoiding oxidative stress at the cellular level [190]. Coupled efficiency of salicylic acid with other plant hormones helps in inducing tolerance to stress environments [191]. Salicylic acid is the endogenous signaling plant hormone that is responsible for providing plants with the ability to resist various pathogens and harmful microbes, which cause disease in the system [192]. Studies suggest that salicylic acid also plays a major role in the production and accumulation of plant secondary metabolites [193]. Mitogen-activated protein kinase (MAPK)-involved pathways also play an important role in biotic stress [194,195]. Studies suggest that the jasmonic acid signaling pathway helps plants to combat stressful environments, and it was also observed that plant secondary metabolite groups such as phytoalexins, terpenoids, and alkaloids are produced in the system, which show prominent anti-microbial activity [196,197].

## 8. Metabolomics Approaches Elucidating the Importance of Secondary Metabolites in Biotic Stress Tolerance

Undoubtedly, the use of metabolomics in tandem with other "omics" techniques, such as transcriptomics and proteomics, could help determine biological or physiological responses to biotic and abiotic stresses [198]. Considering that biosynthesis of secondary metabolites is one of the major ways to resist biotic stress, metabolomics is an emerging approach in understanding the important metabolites in stress tolerance. Metabolomics analysis of biotic stress tolerance has been mostly focused on pathogen attack studies to identify antimicrobial molecules. GC-MS, LC-MS, LC-MS/MS, UHPLC-TOF-MS or CE-MS, MALDI, and NMR, are some of the commonly used methods for metabolite analysis [199].

According to Atanasova-Penichon et al. (2016), a variety of cinnamic acid derivatives, benzoic acid derivatives, flavones, and flavanones have significant contributions to tolerance against fatal Fusarium species and deadly mycotoxin tolerance in cereals [200]. *Burkholderia glumae* influenced the accumulation of phenolic lipids; 5-n-alkylresorcinols (AR) in rice plants at different growth stages were analyzed by Marentes-Culma et al. (2022) via LC-MS characterization [201]. Apart from this, a novel protein, 14–3–3GF14f, was discovered to be involved in *Rhizoctonia solani* tolerance in rice [202]. Recently, the metabolomics analysis of Sorghum plants under *Colletotrichum sublineolum* stress found that alterations in phenylpropanoid and flavonoid pathways resulted in overaccumulation of phenolic compounds such as apigeninidin, phytoalexins, luteolinidin, and related compounds [203]. Phosphoric acid, malic acid, tetrahydroxychalcone, and soluble carbohydrate modifications in Fusarium-infected *Medicago truncatula* plants have been used in breeding programs as metabolic markers for fusarium tolerance varieties [204]. Similarly, the accelerated production of phytoalexins, coumarins, and flavonoids with respect to *Rhizoctonia solani* infection in soybean was also reported [205]. Yang et al. (2020) also found a significant increase in flavonoids, anthocyanin-containing compounds, and secondary metabolite biosynthetic processes upregulating proteins in response to *Tetranychus cinnabarinus* infection in cassava plants [206].

According to Kortbeek et al. (2021), breeding initiatives aimed at selecting cultivars of tomato with increased insect resistance may use acyl sugar metabolites as a target [207]. Overproduction of metabolites from the glycerophospholipid, phenylpropanoid, flavonoid, lignin, fatty acid, and terpenoid biosynthetic pathways was also observed in *Fusarium graminearum* resistant transgenic wheat plants [208]. Similar activations of phenol and flavonoid biosynthetic pathway metabolites were also reported in leguminous plants under different pathogen attacks [209]. However, in yellow mosaic India virus-infected mung bean plants, the metabolomics analysis showed that the main alterations were in primary metabolites such as carbohydrates, amino acids, and organic acids [210].

## 9. Metabolic Engineering in Biotic Stress Resistance Breeding

Plant breeding research is mostly focused on cultivating pest- and disease-resistant variants because the spread of insects or pests due to global climate change raises concerns about the security of the world’s food supply. The modern omics approaches are found effective in elucidating the role of secondary metabolites in pest management at the molecular level. It became simpler to develop pest-resistant organisms attributable to an improved understanding of the molecular mechanisms behind secondary metabolite biosynthesis, storage, and structure. By reducing chemical inputs, metabolic engineering has the potential to alter the amounts of released secondary metabolites and their composition, enabling more sustainable agriculture methods. The production of defensive plant metabolites can be increased, existing biochemical pathways can be changed, or completely new classes of specialized metabolites can be biosynthesized and introduced into recipient plants to augment this chemical resistance to insect herbivory [211].

Because glandular trichomes are thought to be frontline protection against biotic stress agents, one of the primary priorities of pest control studies was to increase the number of trichomes as well as the quality and quantity of secondary metabolites in trichomes. According to Wang et al. (2001), silencing of the P450 hydroxylase gene resulted in increased trichome metabolite production and resistance to aphids in transgenic tobacco plants [212]. Similarly, suppression of the *Amorpha-4,11-diene* synthase (AMS) gene in *Artemisia annua* resulted in the accumulation of the precursors of artemisinin [213]. Transgenic tomatoes that have been modified to have type-IV trichomes on their leaves may be insect resistant. Transgenic tomatoes that have been engineered to have type-IV trichomes, which produce more acyl sugars on their leaves, may be insect resistant [214]. The epidermis-specific transgene expression of sesquiterpene synthase genes from wild tomatoes showed potato aphid resistance in transgenic tomato plants [215]. Recently, Wang et al. (2022) made tomato type-VI glandular trichomes to produce *trans*-chrysanthemic acid using metabolic engineering via coexpressing the *trans*-chrysanthemic-acid pathway-related genes [216].

For disease resistance, phytoalexin can be enhanced or restored using genetic engineering techniques. With this technique, the introduction of a phytoalexin synthesizing gene can be carried out. The stilbene synthase (STS) gene is well known for the production of phytoalexin. Different transformed plant varieties such as tomato, papaya, rice, barley, wheat, tobacco, and alfalfa show resistance against a wide variety of pathogens [183]. In tobacco plants, overexpression of cytokinin showed resistance against the *Phytophthora syringae* pathogen. This is due to the regulation of scopoletin and capsidiol, which are engaged in the process [217]. The transformation of the hairy root of soybean with the ROMT (resveratrol-0-methyltransferase) and AhRS3 (peanut resveratrol synthase 3) genes shows resistance against *Rhizoctonia salami* in transformed plants [218]. A few studies reported that some genes work as a phytoalexin. For example, in rice, overexpression of Rac protein results in the accretion of momilactone phytoalexin and shows resistance against bacterial blight.

Reduced aphid colonization, resisted hornworm feeding, or recruited predatory mites as “bodyguards” against arthropod infestation were seen in transgenic plants that produced elevated quantities of specific terpenoids as a result of the expression of heterologous terpene synthases [219]. The metabolic engineering of *A. thaliana* with berberine via the integration of the BBE gene from *B. lyceum* has been reported to be successful in herbivorous pest management [220]. In addition to this, genetically modified crops are also useful for attracting parasitoids or enemies of pathogens [221,222]. However, inadequate understanding of the biosynthetic pathways of plant-specialized metabolism, inadvertent effects of rerouting plant metabolism, suboptimal transgene design and demonstration procedures, and the stipulation for tissue-specific output of combative substances to ramp up herbivore resistance are all factors that limit current plant genetic engineering strategies [211].

## 10. Exploitation of Secondary Metabolites for Sustainable Pest Management

Current agricultural practices widely use chemical pesticides and insecticides to reduce the crop loss from pests and diseases. The large-scale use of such chemicals results in serious health and environmental issues. New and sustainable crop protection is required due to the world’s fast rising food demand and the escalation of related issues brought on by the effects of climate change. The characteristics of plant secondary metabolites as pest control agents are the foundation of companion farming or push–pull agricultural systems [223]. Companion farming and botanical insecticides are such tools for sustainable pest management.

### 10.1. Companion Farming

Companion farming is the method in which main crops are planted with other crops. They either directly block the unique chemical cues that pests use to locate their hosts or they harbor and maintain particularly potent natural foes of other pests, which helps to reduce pest attack [224]. In companion farming, the plant that attracts the pests from the main crop is known as the trap/pull crop, and the one that produces volatile compounds to repel the pest is known as the push crop [225]. There are many reports that show the success of companion farming in pest management. *Melinis minutiflora*, which is cultivated as an intercrop with maize, offered a “push” by deterring gravid stem borer moths from maize, while other cow feed grasses, such as *Pennisetum purpureum* and *Sorghum vulgare* sudanense, provided the “pull” by releasing large amounts of common plant volatile signals that attracted gravid lepidopteran stem borers when intercropped [226]. The push and pull system with Brassica and marigold reduced pest attacks in the main crop by producing volatiles to attract the enemies of pests of Brassica species [227]. Cheruiyot et al. (2021) reported that the companion farming of maize with forage grasses attracted fall armyworm adults, a major pest of maize [228]. Companion farming, especially the push–pull system, which addresses current constraints, can increase cereal production threefold without seasonal inputs while simultaneously enhancing animal husbandry and providing a number of other benefits, is a viable option for the sustainable intensification of small-holder farming [229]. Table 8 highlights the plants commonly utilized in the practice of companion farming.

### 10.2. Botanical Insecticides

The development of insecticides is currently particularly interesting in the unexplored repository of plant secondary metabolites. Due to their detrimental effects on target pest populations, such as antifeedant, toxic, repellent, fumigant, attractant, molting disturbance, respiratory inhibition, pheromone-based behavioral alterations, oviposition deterrence, and fecundity reduction, these natural compounds are becoming extremely relevant in pest management [237]. Due to their reduced impact on the environment, botanical insecticides are preferred over chemical pesticides. Azadirachtin from neem (*Azadirachta indica*) and pyrethrin from pyrethrum are two examples of botanical molecules having a pesticidal activity that have been successfully identified and commercialized [238].

Numerous studies have found that plant secondary metabolites have the ability to reduce a range of pests, which has been exploited in the preparation of various compositions of botanical pesticides [239]. The formulations of seed oils of *Pongamia pinnata* L., *Pachyrhizus erosus* L., and *Annona squamosa* L. were found effective against the aubergine aphid (*Aphis gossypii* G.) and whitefly as well as the cabbage aphid (*Brevicoryne brassicae* L.) [240]. Several plants, including ryania (*Ryania somnifera*), pyrethrum (*Tanacetum cinerariifolium*), neem (*Azadirachta indica*), sabadilla (*Schoenocaulon officinale*), and tobacco (*Nicotiana tabacum*), are providers of commercially accessible botanical pesticides [238]. Table 9 provides an overview of the botanical insecticides employed in pest management.

## 11. Conclusions and Prospects

Being sessile organisms, plants are prone to various pests and pathogens that challenge their productivity. Secondary metabolites are a diverse class of molecules that are synthesized in plants to protect themselves from herbivores and microbes. These metabolites help to keep us safe from biotic stressors either by indirect or direct defense mechanisms. Apart from the direct effects, such as phytotoxins and phytoalexins, as well as phytoanticipins, some of the secondary metabolites such as volatile compounds are useful in repelling herbivores. Modern metabolomics approaches that have boosted our comprehension of secondary metabolites at the molecular scale have shifted plant breeding approaches to focus on secondary metabolites for biotic stress management. However, as a part of integrated pest management, secondary metabolites have been employed in the development of biopesticides and companion farming. Future research is needed to develop biotic stress-tolerant varieties by improving the quality and quantity of secondary metabolites with the aid of genetic engineering and omics technologies to address global food security. Farmers in developing nations are less inclined to embrace these methods and instead choose to use chemical pesticides. Environmentally friendly alternatives to chemical pesticides, such as plant-based metabolites to control diseases, must be investigated because careless use of these toxic pesticides is ruining our environment.

## Figures and Tables

**Table 1:** List of secondary metabolites with their category and function present in different plants.

Secondary Metabolite	Category	Plant	Function	Reference
Pyrethrins	Monoterpenes	*Chrysanthemum indicum*	Neurotoxic to parasitic wasps, insects, moths, etc.	[20]
Hemigossypol	Sesquiterpenes	*Gossypium hirsutum*	Antifungal	[26]
Gossypol	Diterpene	*Gossypium hirsutum*	Antifungal and antibacterial	[20]
Cucurbitacin-C	Triperpene	*Cucurbita* spps.	Toxic to spider mites	[27]

**Table 3:** List of lectins present in plants with target insects.

Lectin	Plants	Insects	Reference
*Allium sativum leaf lectin*	*Cicer arietinum*, *Nicotiana* spp.	*Aphis craccivora*, Aphids	[45]
Snowdrop lectin	*Triticum aestivum*, *Oryza sativa*, *Arabidopsis* spp.	*Nilaparvata lugens*, Aphids, *Pieris rapae*, *Spodoptera littoralis*	[46]
Nictaba-related lectins NICTABA, PP2	*Nicotiana* spp.	*Manduca sexta*, *Spodoptera littoralis*, *Acyrthosiphon pisum*	[47]
*Arum maculatum tuber lectin*	*Arum maculatum*	*Aphis craccivora*, *Lipaphis erysimi*	[44]
*Bauhinia monandra leaf lectin, Jacalin-like lectins*	*Nicotiana* spp., *Triticum aestivum*	*Anagasta kuehniella*, *Mayetiola destructor*, *Callosobruchus maculates*, *Zabrotes subfasciatus*	[48]

**Table 4:** List of different alkaloids with their examples.

Alkaloid Class	General Information	Example	Reference
Tropane alkaloids	Contains 8-azabicyclo. Present in Solanaceae, Erthroxylaceae, and Convolvulaceae families. They are toxic in nature and are pharmaceutically potent.	Hyoscine, hyoxcyamine, and atropine.	[50]
Benzyl-isoquinoline alkaloid	Pharmacologically active and shows potential against pathogenic infection. Present in Ranunculaceae, Papaveraceae, Menispermaceae, Fumariaceae, and Magnoliaceae families.	Tubocurarine, morphine, berberine, sanguinarine, colchicines, etc.	[21]
Purine alkaloids	Theobromine is obtained by *Camellia ptlophylla* and coffee from *Ilex paraguariensis*, *coffea arabica.*	Theobromine, caffeine	[51]
Simple alkaloids	A simple alkaloid found in *Nicotiana* species which is native to America. Tobacco is eaten, smoked, and chewed. It is also used to kill parasites.	Quinolinic acid, nicotine	[52]
Quinolizidine alkaloids	Toxic in nature. Seeds are a good source of protein. Present in the Leguminosae family.		[53]
Pyrrolizidine alkaloids	Extremely toxic in nature. Fabaceae, Orchidaceae, Asteraceae, and Boraginaceae.	Putrescine, spermidine	[54]

**Table 5:** List of cyanogenic glycosides involved in pest tolerance.

Phytochemical	Plant Source	Target Pest	Reference
Leptin	*Solanum chacoense*	*Leptinotarsa decemlineata*	[55]
α-Chaconine	*Solanum tuberosum*	*Tecia solanivora*	[56]
2/3-Methylbutyronitrile	*Populus trichocarpa*	*Lymantria dispar*	[57]
Dhurrin	*Sorghum bicolor*	*Phyllotreta nemorum*	[58]
α-Solanine	*Solanum tuberosum*	*Tecia solanivora*	[56]
Nicotine	*Nicotiana attenuata*	*Spodoptera exigua*; *Manduca sexta*	[59]

**Table 8:** Plants used in companion farming.

Major Crop	Companion Plants	Enemy	Mechanism	Reference
*Triticum aestivum* L.	*Vigna radiata* L.	*Sitobion avenae*	The odor blends of host and non-host plants affect the host selection of *S. avenae*	[230]
*Triticum aestivum* L.	*Brassica napa* (*Allium sativum*)	*Sitobion avenae*	Attracting natural enemies (lady beetle)	[231]
*Zea mays*	*Desmodium* spp.	*Striga hermonthica* (Del.) Benth.	Producing allelochemicals to suppress the *Striga hermonthica* (Del.) Benth. germination	[232]
*Zea mays*	*Vicia faba*	*Spodoptera frugiperda*	Feeding and repellents	[233]
*Brassica oleracea*	*Brassica nigra*, *Tagetus* spp.	Common brassica pests	Volatiles attracted natural enemies of pests	[227]
Vegetable crops	*Tagetes minuta*, *Artemisia annua*, *Bidens pilosa*, and *Chrysanthemum cinerariaefolium*	*Meloidogyne incognita*	Root volatiles are repellants to nematodes	[234]
*Rubus* species	*Mentha × piperita*	*Drosophila suzukii*	Volatiles act as oviposition deterrent	[235]
*Allium cepa* L.	*Fagopyrum esculentum Moench.*	*Onion thrips*, *Thrips tabaci Lindeman*	Pests are drawn by companion plant volatiles	[236]

**Table 9:** Botanical insecticides used in pest management.

Botanical Insecticide	Secondary Metabolite	Target Organism	References
Garlic aqueous extracts	Sulfur-containing compounds/allicin	Coleoptera, Lepidoptera, and Hemiptera insect species	[241,242]
Neem oil/emulsions	Azadirachtin and limnoids	Blatt dean, Hemiptera, Lepidoptera, and Thysanoptera pests	[243]
*Mentha piperata*	Essential oil	*Callosobruchus maculatus*, flies, lice, moth, and *Tribolium castrum*	[244]
*Ruta chalepensis*	Furocoumarin and quinolone alkaloids	*Spodoptera littoralis*	[245]
*Zingiber zerumbet*	Essential oil	*Lasioderma serricorne*	[239]
*Cephalotaxus sinensis*	Essential oil	*Megoura japonica*, *Plutella xylostella*, and *Sitophilus zeamais*	[246]
*Adenium obesum*	Cardiac glycoside neriifolin	*Monacha obstructa*	[247]
*Periploca sepium Bunge*	Pregnane glycosides	*Armyworm* (*M. separata*)	[248]
